# Pneumorrhachis as a complication of a severe pressure ulcer: An unique case report

**DOI:** 10.1016/j.radcr.2024.02.085

**Published:** 2024-03-21

**Authors:** Rosario Francesco Balzano, Giacomo Fascia, Manuela Montatore, Giuseppe Guglielmi

**Affiliations:** a Radiology Unit, “Dimiccoli” Hospital, Viale Ippocrate 15, 70051, Barletta (BT), Italy; b Department of Clinical and Experimental Medicine, Foggia University School of Medicine, Viale L. Pinto 1, 71122 Foggia, Italy.; c Radiology Unit, “IRCCS Casa Sollievo della Sofferenza” Hospital, Viale Cappuccini 1, 71013 San Giovanni Rotondo, FG, Italy

**Keywords:** Pneumorrhachis, Intraspinal air, Ulcer, Decubitus, Infection, CT, Spinal canal air

## Abstract

Pneumorrhachis is a medical condition that refers to the presence of air within the spinal canal. Many circumstances, including trauma, infection, or medical procedures, might lead to this syndrome.In some cases, pneumorrhachis may not cause any symptoms and can resolve on its own. However, it can also be associated with more severe underlying conditions, such as spinal fractures, spinal infections, or underlying lung pathologies that lead to air escaping into the spinal canal. In this case we report an incidental finding of pneumorrhachis in a patient who came to our attention for suspected sepsis.

## Introduction

Pneumorrhachis, the phenomenon of intraspinal air, is caused by several aetiologies: there are 3 groups of causes, iatrogenic, traumatic, and nontraumatic. In some circumstances, it is not possible to recognize the mechanism behind the phenomenon. The air may be present throughout the canal spine or only at the area of the injury [1]. Usually the Pneumorrhachis is asymptomatic, and the imaging is the only method to diagnose it. This phenomenon is as rare as it is difficult to control and requires multidisciplinary management [2].

## Case presentation

**Fig. 1:**
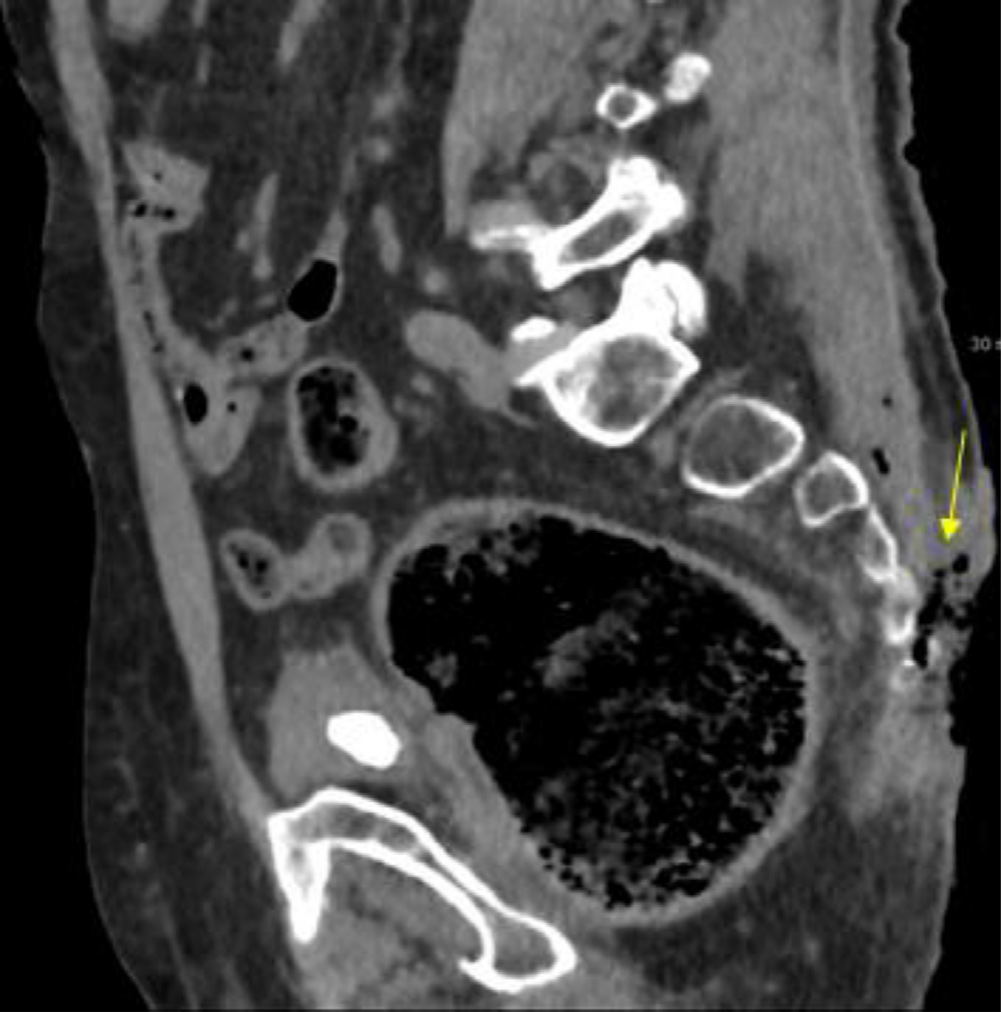
Decubitus ulcer viewed in sagittal plane: loss of skin and muscle tissue (arrow).

An 84-years-old male patient was admitted to the emergency room for fever unresponsive to acetaminophen, mental confusion, and aphasia. He was a bedridden patient with a positive history for chronic renal failure. In the Emergency Room, a bedsore was documented in the sacrum-coccyx region (Fig. 1).

The diagnostic suspicion was sepsis, and a CT of the brain and abdomen was performed, with multiplanar (MPR) reconstructions, to exclude a neurologic disease, or a bleeding and to document a possible septic outbreak.

**Fig. 2:**
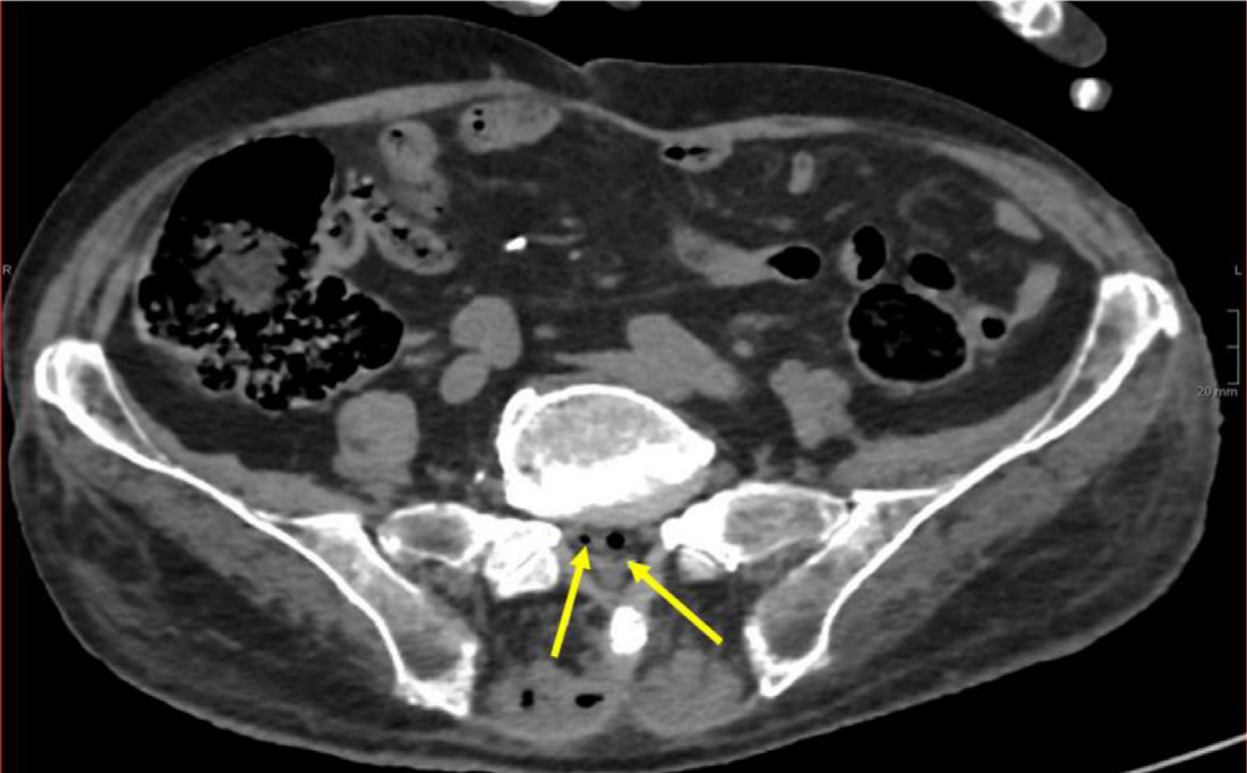
Pneumorrhachis at the sacral level in axial plane (yellow arrows).

**Fig. 3:**
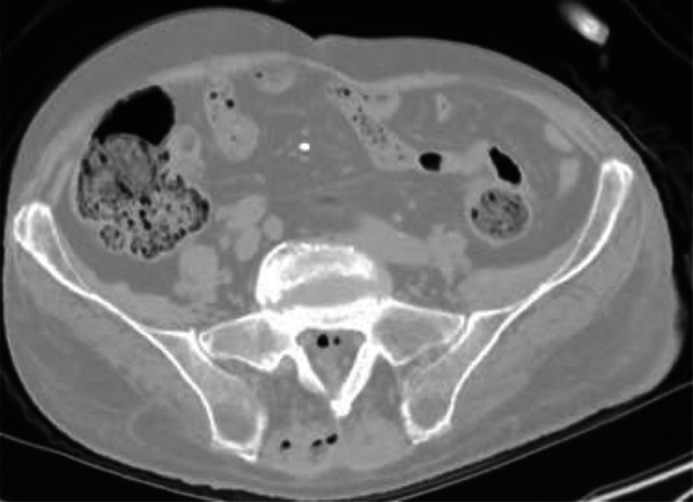
Pneumorrhachis at the sacral level in axial plane, lung window: air in the spinal canal and soft tissue.

On CT examination, no radiological findings, consistent with the clinical question, were detected in the internal parenchymal organs; instead, it showed loss of cutaneous and subcutaneous tissue in the sacral region with evidence of emphysema and stranding of subcutaneous adipose tissue, as well as bone changes in the last sacral and first coccygeal vertebrae, as a condition of osteomyelitis (Figs. 2 and 3).

**Fig. 4:**
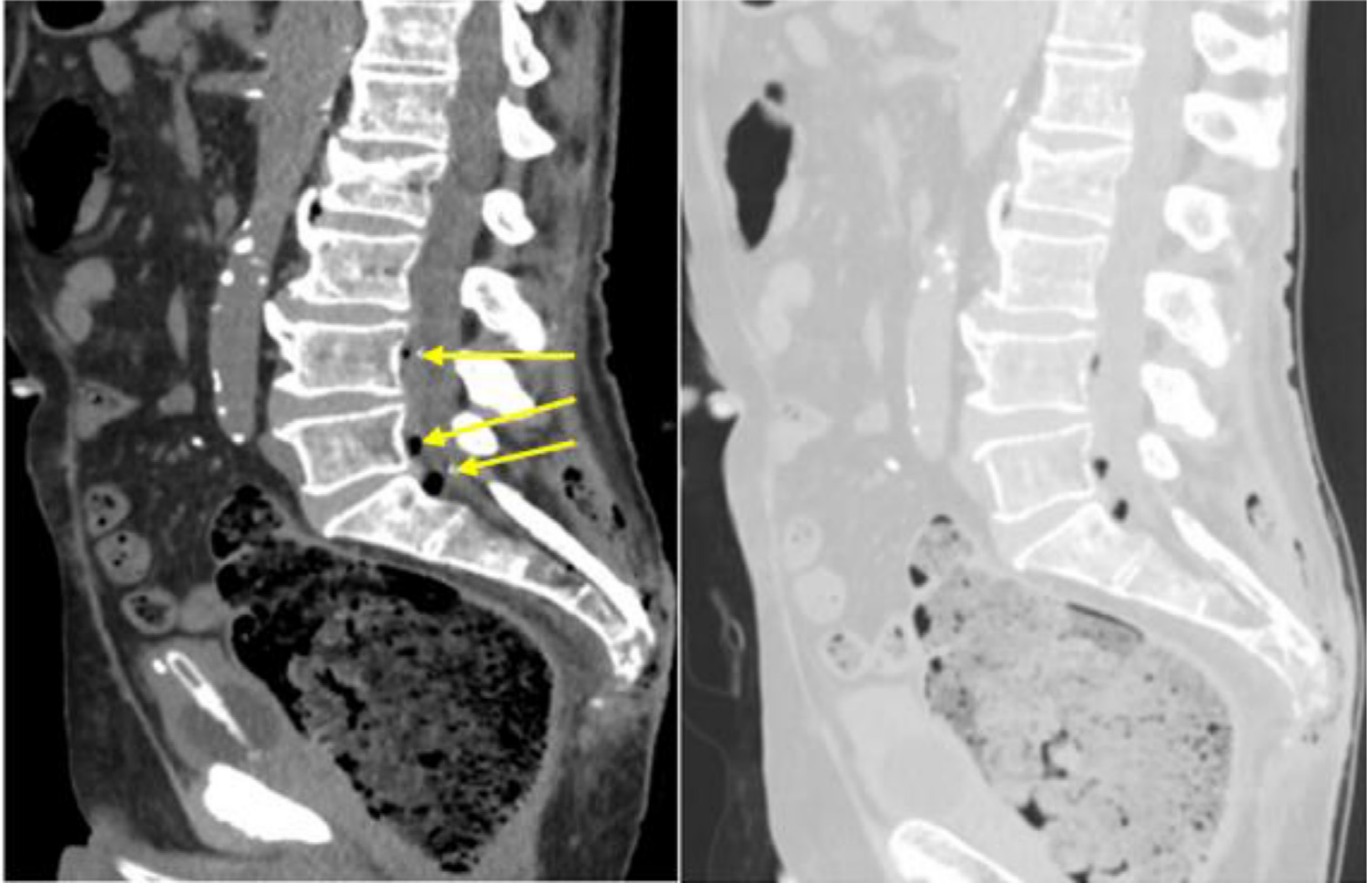
Pneumorrhachis viewed in sagittal plane (yellow arrows) without and with lung window.

Moreover, lung window setting images provided evidence of air in the anterior epidural section of the spinal canal at the level L3-S1, a phenomenon called pneumorrhachis (Fig. 4).

## Discussion

An uncommon occurrence called pneumorrhachis is defined by air being present in the spinal canal. Numerous things, including trauma, infections, and medical treatments, can cause it. The air can enter the spinal canal through several routes, including the bloodstream, the intervertebral foramina, or directly from a nearby injured tissues.

Decubitus ulcers or bedsores, occur over bony prominences as a result of prolonged pressure, friction, or shear forces.

They are common in individuals who are bedridden, use wheelchairs, or have limited mobility. Pneumorrhachis is a rare complication of pressure ulcers, with only few reported cases in the literature [4], [5], [6]. When both pneumorrhachis and decubitus ulcers occur concurrently, it is crucial to manage both conditions to prevent any complications and ensure the best possible outcome for the patient [3,7]. Treatment typically involves addressing the underlying causes of both conditions. Regular repositioning, proper wound care, and adequate nutrition are vital components of managing pneumorrhachis and decubitus ulcers [[8], [9]].

Plain radiographs may reveal the presence of air within the spinal canal, although they may not provide detailed information about the extent or cause of the condition. CT scans are highly effective in visualizing the spinal canal and identifying the presence of air within the spinal canal and can also help identify any associated spinal cord injuries or fractures [10]. MRI is particularly useful in providing detailed images of soft tissues and can help evaluate any associated spinal cord compression or other abnormalities [1]; MRI can also aid in determining the underlying cause of pneumorrhachis, such as a spinal cord injury or infection.

## Conclusion

Due to the rarity and varied causes pneumorrhachis, there are no standard guidelines for its management. If an infectious process is suspected, it is important to perform lumbar puncture as soon as possible to culture the responsible organism. It is necessary to promptly start a broad-spectrum antibiotic treatment that can cross the blood-brain barrier and ensure coverage for anaerobic bacteria. It is important to know about the significance and implication of gas in the spinal canal in the setting of sepsis. When evaluating septic patients with spinal osteomyelitis, a secondary meningitis, a possible consequence of the direct extension of the disease into the spinal canal, must be ruled out. CT scans allow to detect all cases of pneumorrhachis, even the rarest, infectious ones.

## Authors contribution

The authors confirm contribution to the paper as follows: study conception and design: B. R. F. Author F. G. Author; data collection: M. M. Author; analysis and interpretation of results: M. M. Author F. G. Author; draft manuscript preparation: B.R.F. Author, F.G. Author. G.G. Author. All authors reviewed the results and approved the final version of the manuscript.

## Patient consent

Written consent was obtained from the patient for publication of relevant medical information within the manuscript.
